# Absence of *Plasmodium inui* and *Plasmodium cynomolgi,* but detection of *Plasmodium knowlesi* and *Plasmodium vivax* infections in asymptomatic humans in the Betong division of Sarawak, Malaysian Borneo

**DOI:** 10.1186/s12936-017-2064-9

**Published:** 2017-10-17

**Authors:** Angela Siner, Sze-Tze Liew, Khamisah Abdul Kadir, Dayang Shuaisah Awang Mohamad, Felicia Kavita Thomas, Mohammad Zulkarnaen, Balbir Singh

**Affiliations:** Malaria Research Centre, Faculty of Medicine and Health Sciences, Universiti Malaysia Sarawak, 94300 Kota Samarahan, Sarawak Malaysia

**Keywords:** *Plasmodium knowlesi*, Malaria, Asymptomatic, Submicroscopic

## Abstract

**Background:**

*Plasmodium knowlesi,* a simian malaria parasite, has become the main cause of malaria in Sarawak, Malaysian Borneo. Epidemiological data on malaria for Sarawak has been derived solely from hospitalized patients, and more accurate epidemiological data on malaria is necessary. Therefore, a longitudinal study of communities affected by knowlesi malaria was undertaken.

**Methods:**

A total of 3002 blood samples on filter paper were collected from 555 inhabitants of 8 longhouses with recently reported knowlesi malaria cases in the Betong Division of Sarawak, Malaysian Borneo. Each longhouse was visited bimonthly for a total of 10 times during a 21-month study period (Jan 2014–Oct 2015). DNA extracted from blood spots were examined by a nested PCR assay for *Plasmodium* and positive samples were then examined by nested PCR assays for *Plasmodium falciparum*, *Plasmodium vivax*, *Plasmodium malariae*, *Plasmodium ovale*, *Plasmodium knowlesi*, *Plasmodium cynomolgi* and *Plasmodium inui*. Blood films of samples positive by PCR were also examined by microscopy.

**Results:**

Genus-specific PCR assay detected *Plasmodium* DNA in 9 out of 3002 samples. Species-specific PCR identified 7 *P. knowlesi* and one *P. vivax*. Malaria parasites were observed in 5 thick blood films of the PCR positive samples. No parasites were observed in blood films from one *knowlesi*-, one *vivax*- and the genus-positive samples. Only one of 7 *P. knowlesi*-infected individual was febrile and had sought medical treatment at Betong Hospital the day after sampling. The 6 *knowlesi*-, one *vivax*- and one *Plasmodium*-infected individuals were afebrile and did not seek any medical treatment.

**Conclusions:**

Asymptomatic human *P. knowlesi* and *P. vivax* malaria infections, but not *P. cynomolgi* and *P. inui* infections, are occurring within communities affected with malaria.

## Background

Prior to 2004, human malarias were thought to be caused by four species of *Plasmodium*; *Plasmodium falciparum*, *Plasmodium vivax*, *Plasmodium ovale* and *Plasmodium malariae*. Discovery of a large number of *Plasmodium knowlesi* cases in the Kapit division of Sarawak [1] led to the inclusion of *P. knowlesi*, a simian malaria parasite [2], as the fifth cause of malaria in humans [3]. Morphological similarities between *P. knowlesi* and *P. malariae*, when examined by microscopy, had led to *P. knowlesi* being misdiagnosed mainly as *P. malariae* [4]. *Plasmodium knowlesi* infections have been reported in other states in Malaysia [5–9] and in other Southeast Asian countries, including Thailand [10, 11], Philippines [12], Singapore [13], Myanmar [14], Indonesia [15], Cambodia [16] and Vietnam [17].

*Plasmodium knowlesi* is the most common cause of malaria in Sarawak. Epidemiology data for 2014–2015 showed that *P. knowlesi* was responsible for 82.9% of the 2174 malaria cases diagnosed in Sarawak [18]. Studies over the past 10 years in Sarawak by the Malaria Research Centre, UNIMAS, using nested PCR assays [1, 19], have confirmed 890 cases of *P. knowlesi* and only 6 cases of *P. malariae* [20]. Since all 6 patients were Sarawakians who had been working in logging camps overseas in malaria endemic countries and had recently returned to Sarawak, it indicated that these infections were acquired overseas and there were no indigenous cases of *P. malariae* in Sarawak.

Human infections with *P. knowlesi* cause a wide spectrum of disease, including fatal infections [3, 5, 21]. Knowlesi malaria induced by mosquito bites under laboratory conditions with the same *P. knowlesi* strain produced varying outcomes in humans that included refractory individuals, those that self cured, and others that required anti-malarial treatment as their infections were deemed severe [22, 23]. Asymptomatic malaria infections have been described for *P. falciparum* and *P. vivax*. Although all *Plasmodium* species can cause asymptomatic malaria, there have not been many reports of asymptomatic *P. malariae* [24] and *P. ovale* [25] infections. *Plasmodium malariae* infection may be asymptomatic or cause only mild symptoms for many years after the initial infection [24]. Detection of these asymptomatic carriers by highly sensitive molecular detection assays has provided a greater understanding of the epidemiology of malaria [26, 27]. Studies on *P. knowlesi* in Sarawak have been solely hospital-based investigations [1, 5, 8, 9, 21], but there have been reports of asymptomatic *P. knowlesi* infections in Vietnam [28] and more recently in Sabah, Malaysian Borneo [29]. In the Vietnam study, species-specific SSU rRNA nested PCR screen of 95 randomly selected *P. malariae* samples by PCR revealed 5 to be mixed infections with *P. knowlesi*. All of them, including their family members, were asymptomatic at sampling time [28]. In the Sabah study, 1147 samples were collected from randomly selected villagers residing at same village as the microscopy-positive patients as well as members of the patients’ and the selected villager’s households. *P. knowlesi* was detected in 20/1147 (1.7%) samples screened by SSU rRNA nested PCR assays. The use of cytochrome *b* nested PCR, SSU rRNA real time PCR and chromosome 3 plasmepsin real time PCR resulted in the detection of more *P. knowlesi* positive samples: 67/372 (18%), 71/335 (21.9%) and 60/289 (20.7%), respectively. Only one of the 1147 asymptomatic individuals was positive by microscopy [29].

*Plasmodium knowlesi* is typically found in nature in long-tailed and pig-tailed macaques [23]. Other simian malaria parasites, such as *Plasmodium cynomolgi* and *Plasmodium inui* that infect these macaques, have also been shown to be capable of inducing malaria in humans through mosquito bites under experimental conditions [23] and there has been one case of a natural infection of a woman by *P. cynomolgi* in Peninsular Malaysia [30]. Both *P. cynomolgi* and *P. inu*i have been described in wild macaques in Sarawak [31]. Since these parasites occur in the same reservoir host as *P. knowlesi*, there is a strong possibility that natural infections in humans of these simian malaria parasites could occur in Sarawak. However, since *P. inui* is morphologically identical with *P. malariae* and *P. cynomolgi* is morphologically similar to *P. vivax* by microscopy [23], these infections have probably been identified by routine microscopy as *P. malariae* or *P. vivax*. Furthermore, human infections with *P. inui* and *P. cynomolgi* largely resulted in mild infections, which would not normally require hospitalization [23]. The recent development of molecular detection methods for simian malaria parasites [31], affords the possibility of examining human samples for simian malaria parasites other than *P. knowlesi*. Studies utilizing these molecular detection assays on communities living close to the forest fringe, such as those in Betong, would indicate whether other simian malaria parasites are being transmitted to humans. Questions relating to whether there are asymptomatic infections, whether there is clustering of cases within longhouse communities and whether there is human-to-human transmission can be answered by conducting longitudinal studies in these communities utilizing molecular detection tools.

In this longitudinal study, dried blood spots from individuals living within communities with reported malaria cases were repeatedly sampled for the presence of malaria parasites. These longhouse communities were in the Betong Division, one of the 12 administrative divisions in Sarawak, in which 161 knowlesi malaria cases had been reported in the 3 years preceding the initiation of this study [18].

## Methods

### Study population and blood sample collection

**Fig. 1:**
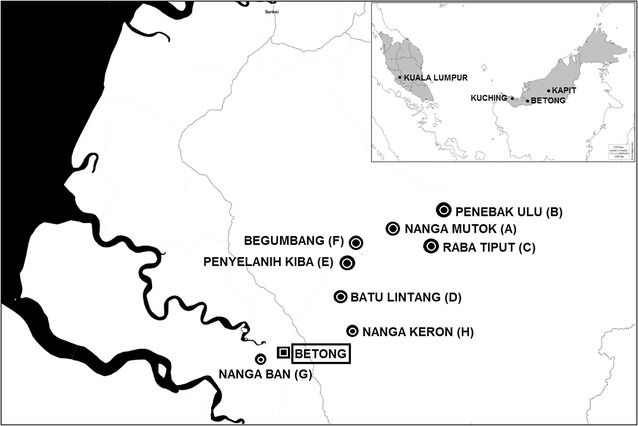
Global Positioning System (GPS) coordinates and study identification (ID) of the 8 longhouses selected in this study: Nanga Mutok (A, 1˚36′18.1″N, 111˚40′24.7″E, Penebak Ulu (B, 1˚39′9.2″N, 111˚44′15.3″E), Raba Tiput (C, 1˚35′8.2″N, 111˚42′7.5″E), Batu Lintang (D, 1˚30′24.3″N, 111˚36′11.0″E), Begumbang (E, 1˚34′25.4″N, 111˚38′3.4″E), Penyelanih Kiba (F, 1˚33′7.21″N, 111˚37′19.07″E), Nanga Ban (G, 1˚23′46.9″N, 111˚31′4.1″E) and Nanga Keron (H, 1˚27′11.8″N, 111˚37′54.6″E)

Participants were recruited from 8 longhouses with residents having a history of recent admission with knowlesi malaria at Betong hospital (Fig. 1). A review of malaria cases in the Betong Division, between 2011 and 2013, identified the following longhouses with a total of 33 knowlesi malaria cases: Nanga Mutok (10 cases), Penebak Ulu (2 cases), Raba Tiput (6 cases), Batu Lintang (1 case), Penyelanih Kiba (2 cases), Begumbang (3 cases), Nanga Ban (1 case) and Nanga Keron (8 cases). All longhouses could be accessed by roads, including during the rainy season. Finger-prick blood samples were taken from each person after informed consent/assent had been obtained. Approximately 30 μl of blood was spotted onto Whatman 3 M filter papers (GE Healthcare & Life science, U.K.) and left to dry at room temperature before being transported to the Malaria Research Centre (MRC) for DNA extraction and molecular detection. Thick blood films were also prepared and transported to the MRC for examination by microscopy. Consenting individuals were sampled bi-monthly, from January 2014 until October 2015. Ethical approval was obtained from the Medical Ethics Committee, Faculty of Medicine and Health Sciences, Universiti Malaysia Sarawak and the Medical Research and Ethics Committee, Ministry of Health Malaysia. The Betong Divisional Medical Office was informed of all the individuals who were PCR-positive for initiation of anti-malarial treatment.

Study participants were asked of their activities within a week prior to the blood collection whether they farmed, hunted or fished and also whether they had recently seen monkeys while farming, hunting or fishing. They were also asked whether they felt unwell or feverish on the day of the blood collection. In addition, temporal artery temperature by Thermoflash^®^ LX-26 (Visiomed, France) was also checked. However, this was only done when it was requisitioned in October of 2014 (5th sample collection).

### DNA extractions and nested PCR assays

**Fig. 2:**
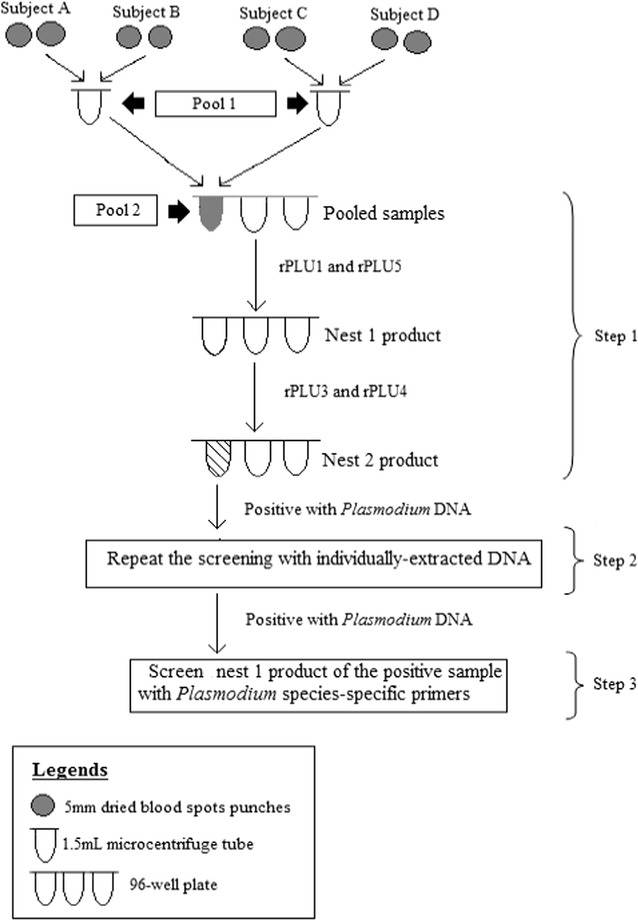
Diagram summarising the pooled strategy used for the screening of DNA extracted from the dried blood spots collected during the first 5 collections

Dried blood spots (DBS) collected between January 2014 and February 2015 (sample collection number 1–7) was extracted based on the pooled strategy of Hsiang [41, 42]. Briefly, four punch holes from two different individuals were put in one tube and DNA was extracted by the Chelex InstaGene™ (Bio-Rad Laboratories, Hercules CA, USA) method as described previously [19] in order to make up DNA samples in Pool 1 (Fig. 2). Next, DNA from two different tubes in Pool 1 was combined in a tube in order to create Pool 2. As shown in Fig. 2, each tube in Pool 2 now contained 4 samples extracted from 4 different individuals.

Detection for the small subunit ribosomal RNA (SSU rRNA) gene of *Plasmodium* was done as previously described [19, 32]. The first step of this three-stepped pooled screening strategy was to screen using the nested PCR assay for the genus *Plasmodium*. Next, DNA from each sample that made up a genus-positive Pool 2 (e.g.: blood spot from individuals A, B, C and D) was re-extracted as described previously and tested separately for *Plasmodium* DNA using the nested PCR assay for genus. Lastly, genus positive samples from step two were screened by species-specific nested PCR assays for *P. falciparum*, *P. vivax*, *P. ovale*, *P. malariae, P. knowlesi, P. cynomolgi* and *P. inui*. As summarized in Fig. 2, pooled DNA samples were amplified with the primers rPLU1 and rPLU5 (nest 1 genus assay) and were then screened using rPLU3 and rPLU4 (nest 2 genus assay). Next, DNA from each of the DBS that made up this *Plasmodium* positive pool were re-extracted and re-screened by nested PCR assay for genus. The nest 1 product from the genus assay, that was detected as containing *Plasmodium* DNA, would then be used as the template for the screen with species-specific primers for *P. falciparum*, *P. vivax*, *P. malariae*, *P. ovale* [19], *P. knowlesi* [1], *P. cynomolgi* and *P. inui* [31].

Positive control dried blood spots, made from 30 μl ring-form cultured *P. falciparum* clone 3D7 parasites diluted with uninfected whole blood to produce parasite densities ranging from 10 to 10,000 parasite per microlitre of blood, were included in the DNA extractions of blood spots taken from the longhouses. Negative control dried blood spots, made from 30 μl uninfected whole blood spotted onto filter papers, were also included in these extractions.

### Staining and examination of blood films

Thick blood films from samples positive for malaria infection by nested PCR assays were stained with 3% Giemsa for 45 min, air dried, mounted using Eukitt^®^ Quick-hardening mounting medium (Sigma-Aldrich, Germany) and examined by microscopy to observe for parasites and the parasite life cycle stage. The number of parasites in 1 μl of blood was calculated based on the formula below [33]:

$${\text{Parasitaemia }}({\text{p/}}\upmu {\text{l}}) = \frac{\text{Number of parasites}}{\text{Number of white blood cells}} \times \, 8000$$

Photos of malaria parasites observed were captured using a 500 megapixel colour CCD camera (model DP21) and Cell^B software (Olympus, America).

### Sequencing and phylogenetic analysis of the SSU rRNA gene

All samples positive for *Plasmodium* DNA were selected for cloning and sequencing of the SSU rRNA gene as previously described [1, 4] with slight modifications. Briefly, the product of the nest 1 genus assay of InstaGene™-extracted DNA was used as the template to generate Phusion^®^ (Finnzymes, Espoo, Finland) blunt-end PCR products using rPLU5 and rPLU6 as the forward and reverse primers. Thermocycler (ProFlex™ PCR system by Applied Biosystem, USA) parameters were as follows: one cycle of initial denaturation at 98 °C for 30 s; 35 cycles of denaturation (98 °C, 7 s), annealing (63 °C, 20 s) and, extension (72 °C, 17 s); one cycle of final extension (72 °C, 10 min). The 1150 bp amplicon produced was visualized on a 1% agarose gel before plasmid ligation and cloning using the Zero Blunt^®^ TOPO^®^ PCR Cloning Kit (Invitrogen, Carlsbad CA, USA) as per manufacturer’s instructions. The presence of the correct gene insert was checked by extracting the plasmid using PureLink™ Quick Plasmid Miniprep (Invitrogen, Carlsbad CA, USA) followed by an *Eco*R I (New England Biolabs, USA) digestion and visualization on a 1% agarose gel. The plasmid DNA containing the 1150 bp gene insert was sequenced using the ABI Big Dye^®^ Terminator Cycle Sequencing Kit (Applied Biosystem, USA) with M13F (forward primer) and M13R (reverse primer). The forward and reverse primer sequences were removed using SeqMan (DNASTAR, Madison, Wisconsin USA) and put into NCBI BLAST^®^ in order to identify the sequence based on its similarity with the other sequences available in the GenBank database. Next, MegAlign (DNASTAR, Madison, Wisconsin USA) was used to align the partial sequences with other *Plasmodium* sequences in GenBank using the Clustal W method. Phylogenetic trees were then constructed using the Neighbour-Joining (NJ) method using MEGA 5.05 [34] with bootstrap percentage based on 1000 replications. All sequences generated were also submitted to GenBank.

## Results

Each of the eight longhouses was visited for a total of 10 times during the 21-month study period (Table 1). A total of 3002 dried blood spots (DBS) and thick blood films were collected from 555 individuals, in which 289 of the recruited participants (52%) and 1697 of the samples collected (56.52%) were from adults. Although the number of study participants started to reduce from the 5th sampling trip, 257 DBS were collected in three out of the 6 remaining sampling trips (Table 1). Females made up 288 of the recruited subjects (52%) and 2642 of the samples collected (88%).

**Table 1:** Total number of longhouses visited, repeat donors, newly recruited donors and blood samples collected for each sampling trip

Sampling number	1	2	3	4	5	6	7	8	9	10	11	Total
Number of longhouses visited	5	8	8	8	8	8	8	7	8	8	4	
Number of repeat donors^a^	–	194	284	265	310	269	252	255	250	256	112	2447
Number of newly recruited donors^b^	256	155	42	39	28	19	5	2	4	1	4	555
Total number of blood samples collected^a, b^	256	349	326	304	338	288	257	257	254	257	116	3002

^a^Total number of repeat donors

^b^Total number of newly recruited subjects

One hundred thirty-two of the 267 adult males (49.4%) recruited in this study reported participating in farming or hunting activities while the rest spent most their time at school or remained within the vicinity of the longhouse. None of the adult females reported participating in hunting activities. Among the adults who farmed, all reported either seeing or hearing monkeys at their farms or that their crops had been eaten, presumably by these monkeys. Those who hunted reported that they went hunting in highly forested areas.

**Fig. 3:**
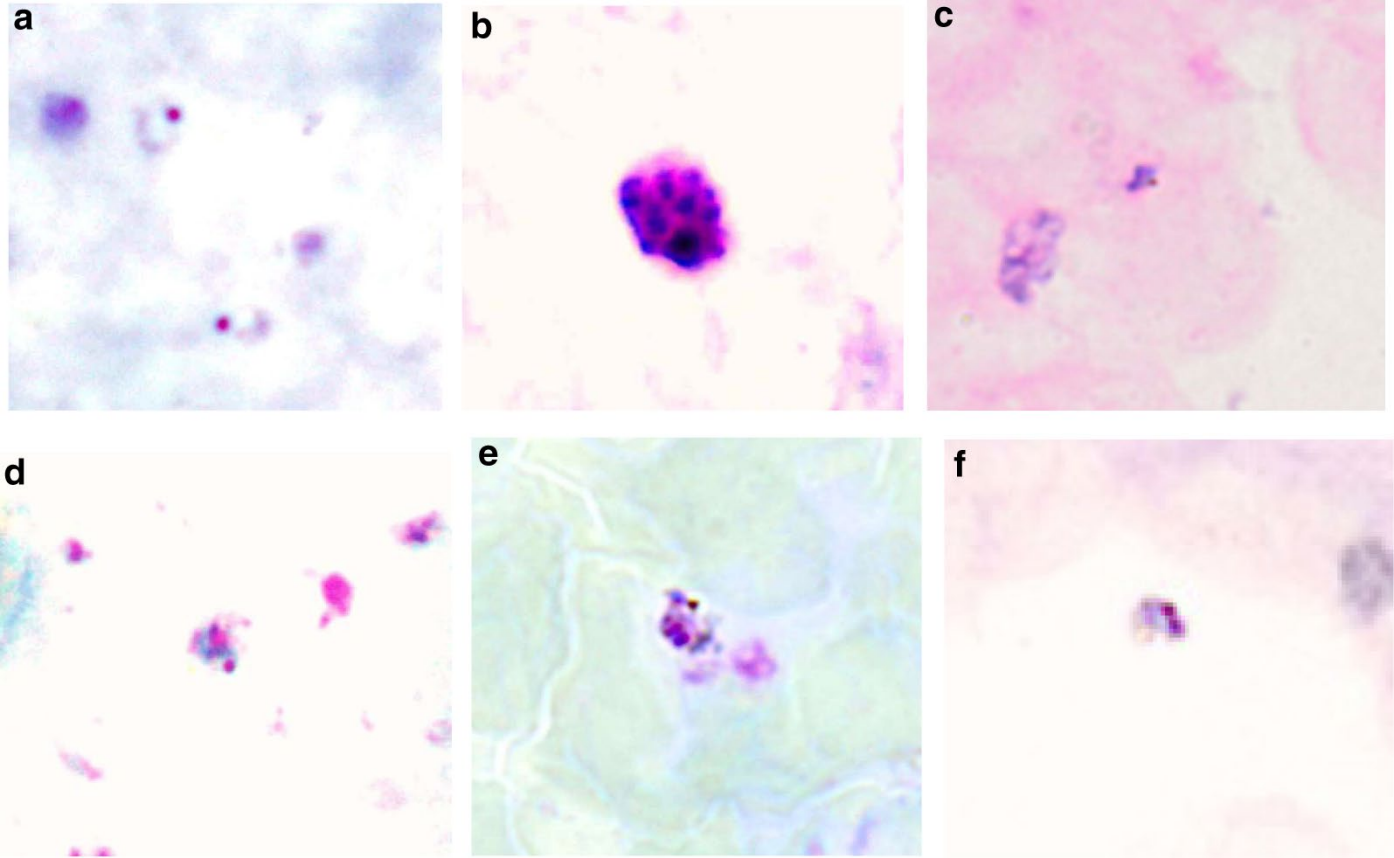
Representative photographs of *P. knowlesi* life stages observed in Giemsa-stained thick blood films from 5 persons: **a**, **b** from A008E; **c** from D001B, **d** from C028C, **e** from C031C and **f** from H011B. With the exception of the photograph labeled **b** (schizont stage), only the ring form of the parasite was observed (photographs labeled **a**–**f**)

A pooled DNA strategy was used to screen 2118 of the 3002 DBS collected between January 2014 and February 2015 (sample trip numbers 1–7, Table 2). Eight of 2118 samples (0.37%) were positive for *Plasmodium* DNA. Screening with species specific primers identified seven *P. knowlesi* infections and one *P. vivax* infection. Since very few cases had been detected using the pooled DNA strategy, DNA from each of the 884 DBS collected between April and October of 2015 were screened individually (non-pooled DNA screening method) in which one sample was detected as positive for Plasmodium DNA (H033, Table 2). However, the *Plasmodium* species for sample H033 could not be determined by the species-specific nested PCR assays (Table 3). The median age of these 7 *P.* knowlesi positive subjects was 45 years, with 5 of them above the age of 60. Of the seven *P. knowlesi* infected subjects, 6 were males. The *P. vivax*-infected individual was a male, while the *Plasmodium* positive sample was from a female subject. Except for sample A008, none of those listed in Table 3 were febrile and none of them sought medical treatment at Betong Hospital. Microscopic examination of the thick blood films from all 9 subjects revealed malaria parasites in 5 of them: A008, C024, C031, D001 and H011 (Fig. 3). In this study, no one was infected on more than one occasion, none lived in the same household and none of their family members were found to be infected (Table 3).

**Table 2:** Nested PCR assays using *Plasmodium* genus and species-specific primers

Sample trip number	Number samples tested	Pool 2^a^ samples	Pool 1^b^ samples	Genus positive only^c^	Species^d^						
					*Pk*	*Pf*	*Pm*	*Po*	*Pv*	*Pin*	*Pcy*
1	256	5	3	0	0	0	0	0	0	0	0
2	349	3	3	0	3	0	0	0	0	0	0
3	326	4	4	0	2	0	0	0	1	0	0
4	304	5	1	0	0	0	0	0	0	0	0
5	338	3	5	0	2	0	0	0	0	0	0
6	288	0	0	0	0	0	0	0	0	0	0
7	257	0	0	0	0	0	0	0	0	0	0
8	257	ND	ND	1	0	0	0	0	0	0	0
9	254	ND	ND	0	0	0	0	0	0	0	0
10	257	ND	ND	0	0	0	0	0	0	0	0
11	116	ND	ND	0	0	0	0	0	0	0	0
Total	3002	66	86	1	7	0	0	0	1	0	0

*ND* not done

^a^Pool 2 samples contained DNA extracted from 4 persons

^b^Pool 1 samples contained DNA extracted from 2 persons

^c^The number of individually extracted DNA samples which were positive for *Plasmodium* genus-specific primers but negative for all of the *Plasmodium* species-specific primers

^d^The number of samples which were positive for *Plasmodium* species-specific primers: *P. knowlesi*: Pk; *P. falciparum*: Pf; *P. malariae*: Pm; *P. ovale*: Po; *P. vivax*: Pv; *P. inui*: Pin; *P. cynomolgi*: Pcy

**Table 3:** Comparison of nested PCR assay and microscopy results of *Plasmodium*-infected study participants

Study ID	Gender	Age (years)	Occupation	Malaria history (year of infection)	Temperature	Blood collection period	Microscopy (parasitaemia)	Nested PCR
D001	M	65	Farmer	None	Afebrile	March 2014	32 p/μl	*Pk*
H011	M	86	Farmer	None	Afebrile	March 2014	48 p/μl	*Pk*
C028	M	45	Farmer	None	Afebrile	March 2014	48 p/μl	*Pk*
C024	M	35	Farmer	None	Afebrile	June 2014	Negative	*Pk*
C031	M	61	Farmer	None	Afebrile	June 2014	48 p/µl	*Pk*
H030	M	61	Retiree	None	Afebrile	June 2014	Negative	*Pv*
A008^a^	M	24	Farmer	None	Febrile (37.4 °C)^b^	October 2014	3536 p/μl	*Pk*
A011	F	42	Farmer	*Pk* (2013)	Afebrile	October 2014	Negative	*Pk*
H033	F	71	Farmer	None	Afebrile	April 2015	Negative	Genus positive

A, Nanga Mutok; C, Raba Tiput; D, Batu Lintang; H, Nanga Keron; F, female; M, male; p/μl, number of parasites per microlitre of blood; *Pk*, *Plasmodium knowlesi; Pv*, *Plasmodium vivax*

^a^This individual had worked at the farm/went hunting the week prior to sample collection

^b^Body temperature by Thermoflash (Visiomed, France) detection of the temporal artery, which was available from October 2014 onwards

One symptomatic *P. knowlesi* infection was detected by nested PCR assays in this study, a 24-year-old male (A008) who sought medical treatment at Betong Hospital the day after his blood sample had been taken. At the time of blood collection (between 6 and 11 pm), his temporal artery temperature was 37.4 °C, the lowest in the temperature range for fever and he did not report feeling unwell. Microscopic examination of the thick blood film revealed a few ring forms (Fig. 3a) and one schizont (Fig. 3b); with an estimated parasitaemia of 3536 parasites/μl of blood. On follow-up, he reported that he had been clearing land for farming in a forested area the week before the blood collection. He was PCR negative for malaria parasite prior to and preceding this infection.

One asymptomatic *P. vivax* infection from a 61-year-old male (H030) was detected in this study. No parasites were observed in his thick blood film and he did not report feeling unwell at the time of the blood collection. Samples taken prior to and preceding this infection was PCR negative for malaria parasites.

**Fig. 4:**
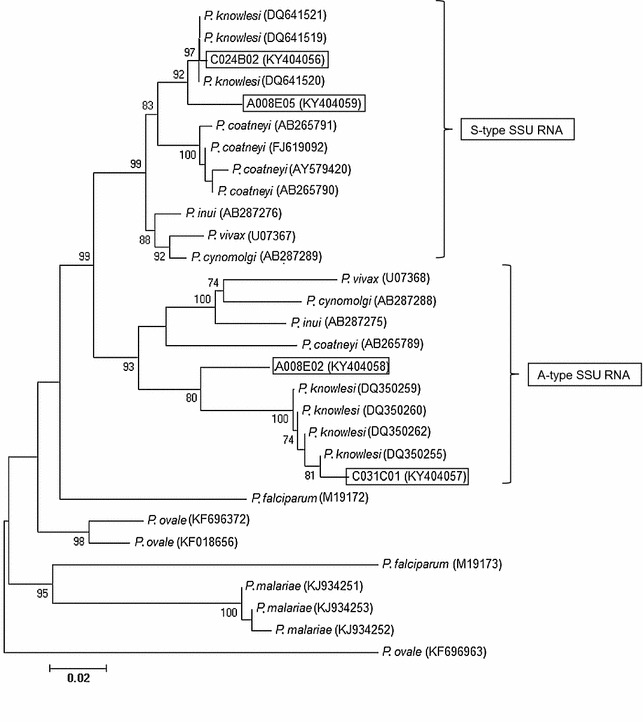
Neighbour-joining tree constructed using partial sequences of SSU rRNA genes of *Plasmodium* species. The sequences generated in the current study are boxed. Bootstrap percentage was based on 1000 replicates and only those above 70% are shown

Parasites infecting A008 and C031 were confirmed as *P. knowlesi* through phylogenetic analysis of the SSU rRNA genes (Fig. 4). However, attempts at sequencing the SSU rRNA gene from samples A011 and C028 were unsuccessful.

## Discussion

The finding of the present study, that sub-microscopic infections were observed in communities that are hypo-endemic for malaria, is consistent with reports by others. In a recent study in Sumatera, Indonesia, 614 of 3731 participants (16.5%) were positive for malaria parasites by microscopy. In comparison, PCR detected parasite DNA in samples from 1169 individuals (31.3%) [15]. In another study in Sumatera, 6 out of 1495 asymptomatic individuals screened were detected as having *P. knowlesi* (n = 1) and *P. vivax* (n = 3) infections [35]. Screening of 638 (in northeast Myanmar) and 1070 (in western Thailand) villagers from these hypo-endemic areas showed that microscopy detected 1.3 and 0.04% infections compared to nested PCR detecting 1.9 and 6.2% as *Plasmodium* positive [36]. A screen of 11,185 samples in western Kenya for *P. falciparum* infections revealed that more false negatives were observed when samples were collected from low malaria transmission as 8% more samples were detected as positive by nested PCR assays compared to microscopy [37]. An average parasite prevalence of 28.4% (in adults) and 25.5% (in children) were reported when 9260 microscopy negative samples from afebrile individuals in Uganda was screened for *P. falciparum* infections by loop-mediated isothermal amplification [38]. More recently, a malaria survey of 845 afebrile schoolchildren in northwest Ethiopia reported 0.95% detection by microscopy compared to 12.7% that were detected as *Plasmodium* positive by real time PCR [39]. Only one of the 1147 (0.08%) afebrile individuals from northeast Sabah of Malaysian Borneo that were screened for *P. knowlesi* was detected as positive by microscopy compared to 20 individuals (1.7%) by nested PCR [29]. Similarly, PCR-based methods detected more malaria infections in two studies done in different provinces in Sumatera: 20/1532 (0.06%) PCR-confirmed *P. knowlesi* infections compared to none by microscopy [35]; 612/3731 (16.4%) *Plasmodium* positive by microscopy compared to 377/3731 (10%) *P. knowlesi* positive by hemi-nested PCR [15]. In the current study, the *P. knowlesi* infections detected were either submicroscopic (n = 2) or had parasitaemia of less than 50 parasites/μl of blood (n = 4).

A higher detection of submicroscopic infections could have been achieved had more sensitive methods or a larger amount of blood and subsequent DNA extraction using commercial kits was undertaken in the current study. One more sample was detected as positive when DNA was prepared from 1708 samples from asymptomatic communities using a commercial DNA extraction kit compared to the standard DNA extraction method [36]. Detection of more *P. vivax* infected samples by nested reverse transcription PCR (182/1005) or nested PCR (24/1005) was attributed to the amount of template used in the assay which was DNA or RNA extracted from whole blood samples [40]. Capture and ligation probe-PCR that used templates extracted from DBS was able to detect only 19/1005 samples screened [40]. In this study, initial DBS screens were done using a high-throughput method in order to reduce the turnaround time for infection detection [41, 42]. The conventional method for screening DBS, in which each DNA sample was assayed individually, was applied in the later part of our study in an order to improve detection of malaria parasites. Compared to a detection limit of 100 parasites per microlitre (p/μl) of blood for the pooled method [41], detection limit of SSU rRNA nested PCR of individual samples was 6 p/μl of blood [19]. However, despite using the more sensitive assay later in our study, only one of the 884 DBS screened was *Plasmodium*-positive that could be related to the decrease in number of reported malaria cases in the Betong division [18]. More infections could have also been detected had the sampling intervals been shorter. A 2-month interval between samplings could increase the risk of missing transient asymptomatic infections that might have resolved spontaneously. Therefore, sampling fewer sites but more frequently might help detecting transient asymptomatic infections within the affected communities.

In contrast to the Sabah study that detected *P. knowlesi* infections in 15–45 year olds [29] and in young children in the Vietnam study [28]; only adults were infected with malaria in our study. With the exception of one of our PCR-positive study participants, all had farms located near forested areas. In addition, all reported seeing long-tailed macaques or signs of their presence at their farms. The location of the farms and presence of long-tailed macaques is in concordance with “forest exposure” as one of the risks for *P. knowlesi* infection [3, 8]. Studies in Sabah [29] and Vietnam [28] found asymptomatic infections to be common in communities affected by *P. knowlesi* infections due to the co-existence with the vector and host [8, 28]. Interestingly, two of the *knowlesi* positive individuals (D001 and H011) reported at follow-up that they mainly stayed within the longhouse compound and that they did not participate in farming, hunting or fishing activities. Both of their longhouses were surrounded by pockets of substantially forested areas. Although this does not preclude undisclosed activities that may bring them near forested areas, a yet-to-be-identified *P. knowlesi* vector that has adapted to dwell within the longhouse compound is a possibility [43].

The DBS from sample H033 could only be determined as *Plasmodium*-infected as the species-specific nested PCR assays failed to produce an amplicon. This could be due to the primer design that was based on either the asexually transcribed (A) or the sexually transcribed (S) forms of *Plasmodium* SSU rRNA gene for the species-specific nested PCR assays [44]. The genus-specific primer pair anneals to both the asexual and sexual forms of the SSU rRNA gene and therefore, nested PCR assays with the genus-specific primers are more sensitive than those with the species-specific assays.

The only asymptomatic *P. vivax* infection was detected in a retired offshore worker who divided his time between his home at the longhouse in Betong and a relative’s home in Miri, a city in the northern region of Sarawak. Although *P. knowlesi* infection is more common in Sabah and Sarawak, 7% of microscopy-confirmed malaria cases reported in 2015 were due to *P. vivax* [18].

Absence of *P. cynomolgi* and *P. inui* infection in asymptomatic individuals in this study supports the rarity in reports of individuals hospitalized with these infections. Only one case of a human naturally infected with *P. cynomolgi* has been reported, which was in Peninsular Malaysia [30], while no natural human infection by *P. inui* has been reported. Human infections by *P. cynomolgi* could be limited by its requirement for select receptors on human RBCs [45]. Lack of suitable vectors could also affect invasion of human RBCs by these species.

There may be people with asymptomatic and sub-microscopic malaria who may be a source of human-to-human transmission. Asymptomatic malaria could be a problem for malaria elimination since most asymptomatic individuals remain untreated and do not seek treatment since they do not develop any signs and symptoms of malaria [46]. Current molecular, epidemiological and entomological evidence suggests that *P. knowlesi* is a zoonosis in Sarawak but human-to-human malaria cannot be ruled out completely. It may be occurring but proving it is going to be difficult because knowlesi malaria transmission is occurring in areas where macaques are found.

## Conclusions

This study showed that asymptomatic *P. knowlesi* and *P. vivax* infections are present in communities with reported cases of *knowlesi* malaria. These infections were either submicroscopic or had very low parasitaemia. None of the asymptomatic *P. vivax* and *P. knowlesi*-infected persons sought medical attention and all were afebrile.

## Abbreviations

DBS: dried blood spot
PCR: polymerase chain reaction
SSU rRNA: small subunit ribosomal RNA
