# Correction to: Absence of *Plasmodium inui* and *Plasmodium cynomolgi*, but detection of *Plasmodium knowlesi* and *Plasmodium vivax* infections in asymptomatic humans in the Betong division of Sarawak, Malaysian Borneo

**DOI:** 10.1186/s12936-017-2093-4

**Published:** 2017-11-06

**Authors:** Angela Siner, Sze-Tze Liew, Khamisah Abdul Kadir, Dayang Shuaisah Awang Mohamad, Felicia Kavita Thomas, Mohammad Zulkarnaen, Balbir Singh

**Affiliations:** Malaria Research Centre, Faculty of Medicine and Health Sciences, Universiti Malaysia Sarawak, 94300 Kota Samarahan, Sarawak Malaysia

## Correction to: Malar J (2017) 16:417 https://doi.org/10.1186/s12936-017-2064-9

After publication of the article [1], it has been brought to our attention that two of the labels on Figure 4 have transposed. The labels “S-type SSU rRNA” and “A-type SSU rRNA” should be in opposite places.
